# Pigment Epithelium-Derived Factor Mediates Autophagy and Apoptosis in Myocardial Hypoxia/Reoxygenation Injury

**DOI:** 10.1371/journal.pone.0156059

**Published:** 2016-05-24

**Authors:** Hsuan-Fu Kuo, Po-Len Liu, Inn-Wen Chong, Yu-Peng Liu, Yung-Hsiang Chen, Po-Ming Ku, Chia-Yang Li, Hsiu-Hua Chen, Hui-Ching Chiang, Chiao-Lin Wang, Huang-Jen Chen, Yen-Chieh Chen, Chong-Chao Hsieh

**Affiliations:** 1 Department of Internal Medicine, Kaohsiung Municipal Ta-Tung Hospital, Kaohsiung Medical University Hospital, Kaohsiung Medical University, Kaohsiung, 801, Taiwan; 2 Department of Respiratory Therapy, College of Medicine, Kaohsiung Medical University, Kaohsiung, 807, Taiwan; 3 Department of Genome Medicine, Kaohsiung Medical University, Kaohsiung, Taiwan; 4 Graduate Institute of Integrated Medicine, College of Chinese Medicine, China Medical University, Taichung, 404, Taiwan; 5 Department of Psychology, College of Medical and Health Science, Asia University, Taichung, 413, Taiwan; 6 Cardiovascular Center, Chi-Mei Hospital, Liouying, Tainan, 736, Taiwan; 7 Chia-Nan University of Pharmacy & Science, Tainan, 717, Taiwan; 8 Division of Cardiovascular Surgery, Department of Surgery, Kaohsiung Medical University Hospital, Kaohsiung, 807, Taiwan; Emory University, UNITED STATES

## Abstract

Pigment epithelium-derived factor (PEDF) is a multifunctional protein that exhibits anti-angiogenic, antitumor, anti-inflammatory, antioxidative, anti-atherogenic, and cardioprotective properties. While it was recently shown that PEDF expression is inhibited under low oxygen conditions, the functional role of PEDF in response to hypoxia/reoxygenation (H/R) remains unclear. The goal of this study was to therefore investigate the influence of PEDF on myocardial H/R injury. For these analyses, PEDF-specific small interfering RNA-expressing and PEDF-expressing lentivirus (PEDF-LV) vectors were utilized to knockdown or stably overexpress PEDF, respectively, within human cardiomyocytes (HCM) *in vitro*. We noted that reactive oxygen species (ROS) play important roles in the induction of cell death pathways, including apoptosis and autophagy in ischemic hearts. Our findings demonstrate that overexpression of PEDF resulted in a significant reduction in ROS production and attenuation of mitochondrial membrane potential depletion under H/R conditions. Furthermore, PEDF inhibited the activation of a two-step apoptotic pathway in which caspase-dependent (caspase-9 and caspase-3) and caspase-independent (apoptosis inducing factor and endonuclease G), which in turn cleaves several crucial substrates including the DNA repair enzyme poly (ADP-ribose) polymerase. Meanwhile, overexpression of PEDF also promoted autophagy, a process that is typically activated in response to H/R. Therefore, these findings suggest that PEDF plays a critical role in preventing H/R injury by modulating anti-oxidant and anti-apoptotic factors and promoting autophagy.

## Introduction

Acute myocardial infarction (AMI) remains the most common cause of cardiac morbidity and mortality worldwide. AMI-induced myocardial hypoxia followed by medical intervention (reperfusion), such as percutaneous transluminal coronary angioplasty or bypass surgery, is an inefficient approach for addressing the symptoms of hypoxia/reoxygenation (H/R) injury, which lead to irreversible damage and complications [1–3]. Previous studies revealed that H/R injury results in increased production of extracellular and mitochondrial reactive oxygen species (ROS), which play a central role in H/R injury-induced cell death [4]. Specifically, H/R-induced ROS induce calcium flux resulting in depression of the mitochondrial membrane potential (Δ*Ψ*_m_) and dysfunction of the mitochondrial permeability transition pore, subsequently leading to cardiomyocyte apoptosis, necrosis, or autophagy. Autophagy (self-digestion) is a process that is necessary for maintaining cellular homeostasis by balancing synthesis and degradation and for protecting cells against damaged or dysfunctional organelles [5]. While the frequency of autophagy remains at a basal level under normal physiological conditions, this process can be induced by cellular stresses such as hypoxia, nutrient deprivation, and growth factor withdrawal [6].

Pigment epithelium-derived factor (PEDF) is a 50-kDa multi-functional glycoprotein that belongs to the structurally homologous serpin superfamily [7]. PEDF is expressed by a variety of cell lines, and has been shown to provide a protective effect against atherosclerosis, myocardial infarction, coronary heart disease, and heart failure via its anti-angiogenesis, antioxidant, and antithrombotic properties [8]. PEDF was also shown to upregulate the expression of B-cell lymphoma 2 (Bcl-2) and promote the survival of cultured cortical neurons by inhibiting oxidative stress and apoptosis [9]. Similarly, a separate study demonstrated that upregulation of Bcl-2 by PEDF leads to inhibition of the ROS-induced nuclear translocation of apoptosis inducing factor (AIF), resulting in prevention of apoptosis both *in vitro* and *in vivo* [10], while Wang *et al*. demonstrated that PEDF reduces hypoxia-induced apoptosis and necrosis in rat myoblast H9c2 cells by inhibiting p53 mitochondrial translocation [11]. Meanwhile, analysis of the regulation of PEDF expression in cancer cells revealed that hypoxic conditions result in reduced PEDF levels via the induction of autophagy and subsequent degradation of PEDF through an autophagic pathway [12]. Hypoxia-mediated reductions in intracellular ROS production and increases in the levels of autophagy or apoptosis in cardiac myocytes have been observed in cardiovascular diseases; however, the functional role of PEDF in the regulation of these processes remains unclear.

Increasing evidence from *in vitro* and *in vivo* studies examining the effects of genetic and pharmacologic manipulation of PEDF suggests that this protein plays a dual role in the heart. While enhancing both PEDF expression and the levels of opportune autophagy under hypoxic conditions can promote survival in response to stress, reducing PEDF expression, thereby inhibiting excessive or long-term upregulation of autophagy, can promote cell death [13]. Therefore, PEDF-mediated modulation of autophagic flux represents a potential future therapeutic target for treating or preventing a variety of cardiovascular diseases. However, numerous questions regarding the role of PEDF in the heart remain unanswered and require further investigation.

Since both apoptosis and autophagy are relevant to myocardial H/R injury, we designed this study to explore the effects of PEDF expression on human cardiac myocytes subjected to H/R injury. Our data suggest that PEDF comprises a novel potential therapeutic target for myocardial H/R injury.

## Materials and Methods

### Cell culture

HCM were obtained from ScienCell Research Laboratories (Carlsbad, CA, USA), and the cells were cultured in cardiac myocyte medium (with 5% of fetal bovine serum, 1% of cardiac myocyte growth supplement, and 1% of penicillin/streptomycin). Cells were cultured in 5% CO_2_ at 37°C, and the culture medium was replaced every 4 to 5 days. With no exception, cell passage was standardized between 3 and 9.

### H/R condition

In order to create a H/R condition, HCM were placed to hypoxia condition. *In vitro* hypoxia model was created by Whitley anaerobic and microaerophilic jar gassing system (don Whitley Scientific). Briefly, confluent beating HCM monolayer cells were exposed in a 0.33% O_2_, 5% CO_2_ and 95% N_2_ gas mixture for 3 (H3) or 24 (H24) h maintained in FBS-free medium (hypoxia), and then the cells were cultured in a 95% air and 5% CO_2_ gas for 6 (R6) h (reoxygenation).

### Measurement of intracellular ROS

HCM were incubated with 5 μM CellROX Deep Red Reagent (C10422, Invitrogen, USA) for 30 min at 37°C, and the medium was removed and the cells were washed twice with phosphate-buffered saline (PBS). Then, HCM were collected and suspended in PBS. The signal was analyzed by FACS using the BD LSRII (BD Bioscience), measuring the fluorescence emission at 590 nm and using excitation at 510 nm.

### Apoptosis assay

An Annexin V assay (Alexa Fluor Annexin V/Dead Cell Apoptosis Kit, Life Technologies, USA) was performed to examine the cell apoptosis. Cells were seeded in 6 cm dish and treated with or without H/R. The medium was removed and the cells were washed in ice-cold PBS, and then the binding buffer was added at a final cell concentration of 1 × 10^5^ cells/ml and incubated with annexin V-FITC for 20 min at 4°C. The medium was removed and the cells were photographed using fluorescence microscope.

### Cell viability analysis

The 3-(4,5-dimethylthiazol-2-yl)-2,5-diphenyltetrazolium bromide (MTT, Sigma, USA) assay was performed as described previously [14]. HCM were incubated with or without H3/R6 or H24/R6 condition, and then the 100 μl of MTT (0.5 mg/ml) was added into the cultured medium. After 4 h of incubation, the medium was removed and cells were washed twice with PBS. The dimethyl sulfoxide (100 μl) was then added to solubilized blue formazan crystals and the absorbance was read at 540 nm using a microplate reader (Multiskan Ex, ThermoLabsystems).

### Quantitative real-time PCR

Total RNA was extracted and then reverse-transcribed using SuperScript^™^ and First-Strand Synthesis System for RT-PCR Kit (Invitrogen). cDNA was used as the standard to quantify the relative content of mRNA by real-time TaqMan-PCR (LightCycler FastStart DNA Master SYBR Green I, Roche) as described previously [15]. The comparative CT (ΔΔCT) method was used with values first normalized to the housekeeping gene β-actin. Human primers (PEDF sense: ATT CCC GAT GAG ATC AGC A; anti-sense: CTT AGG GTC CGA CAT CAT GG; β-actin sense: TCC CTG GAG AAG AGC TAC GA; anti-sense: AGC ACT GTG TTG GCG TAC AG) were obtained from Integrated DNA Technologies (MD Bio).

### Western blotting analysis

Western blot was performed as described previously [15], cells were washed with PBS and lysed in protein lysis buffer (BioRad) containing protease inhibitor. Cytoplasm protein extracts were separated to 10% SDS–PAGE and then transferred to polyvinylidene difluoride membranes for 1h at room temperature. The membranes were incubated overnight at 4°C with primary antibody against PEDF (1:500, Abcam), Beclin 1 (1:500, GeneTex), microtubule-associated protein 1A/1B-light chain 3 (LC3)-I/II (1:500, Santa Cruz), Atg5-Atg12 (1:500, Santa Cruz), caspase-3 (1:500, GeneTex), caspase-9 (1:500, GeneTex), AIF (1:500, GeneTex), endonuclease G (EndoG) (1:500, GeneTex,), poly (ADP-ribose) polymerase (PARP) (1:500, Abcam), α-tubulin (1:1000, Sigma), α-tubulin (1:1000, Sigma), Histone H1 (1:1000, GeneTex), Lamin A/C (1:1000, GeneTex). We used different loading control repeatedly to confirm protein loading between the amounts of each group as equal much and the loading controls have different molecular weights than the proteins of interest. After incubation with appropriate horseradish peroxidase-labeled secondary antibodies for 1 h at room temperature, the protein bands were detected using ECL-Plus reagent (Millipore) and exposure to Biomax MR Film (Kodak, Rochester, NY, USA). Data were quantified using ImageQuant 5.2 (Healthcare Bio-Sciences, Philadelphia, PA, USA). Each bar graph shows the summarized data from separate experiments by densitometry after normalization to loading controls.

### Construction of human PEDF (hPEDF) lentiviral vector

For cloning of hPEDF, total RNA was isolated from HCM, and the cDNA was produced using MMLV reverse transcriptase according to the manufacture’s manual (Invitrogen, Carlsbad, CA, USA). The hPEDF cDNA (GenBank accession number AF400442.1) was amplified by PCR using the following primers: forward: 5’-GTC CTA CTA GTG CCA CCA TGC AGG CCC TGG TGC TAC TC-3’; reverse: 5’-CTT ACG CGG CCG CTT AGG GGC CCC TGG GGT CCA-3’. The hPEDF cDNA was cloned into pCR2.1-TOPO vector (Invitrogen), and subcloned into pLEX-MCS lentiviral vector between the BamHI and XhoI restriction enzyme sites.

### Lentivirus production and transduction

The hPEDF and pLEX-MCS vector-only lentivirus particles were prepared by co-transfection with the packaging plasmid SPAX2 and the envelope plasmid MD2G into HEK293T cells using calcium phosphate precipitation method. The lentivirus-containing medium was collected at 48 and 72 h post-transfection. After centrifugation, the lentivirus medium was filtered using a 0.45 μm syringe filter. For lentivirus transduction, the primary cells and cell lines were incubated with the 1:1 combination of culture medium and lentivirus medium containing 5 mg/ml polybrene. After a 4-h incubation, the medium was replaced by complete culture medium and allowed the cell growth for 3 days. For the stable clone selection, the lentivirus-transduced cells were treated with 1 mg/ml puromycine. The transfection did not significantly influence cell viability (>90%).

### Measurement of *ΔΨm*

H/R-induced the *ΔΨm* change were measured by JC-1 mitochondrial membrane potential detection kit (Cell technology, Fremont, CA, USA). JC-1 (5,5’,6,6’-tetrachloro-1,1’,3,3’-tetraethylbenzimidazolylcarbocyanine iodide) exists as a monomer (low potential; <140mv) in the cytosol (green) and also accumulates as aggregates (high potential) in the mitochondria which stain red as described previously [16]. HCM were treated to H/R, and then incubated with 2.5 μg/ml JC-1 at 37°C for 15 minutes. The cells were then examined and photographed using fluorescence microscope and quantification by flow cytometry analysis. Mitochondria containing red JC-1 aggregates in healthy cells are detectable in the FL2 channel, and green JC-1 monomers in apoptotic cells are detectable in the FITC channel (FL1).

### PEDF silencing in cells

PEDF siRNA was obtained from Santa Cruze (SC-40947; CA, USA). HCM were cultured in 6-well plate for 24h and then transiently transfected with 20 nM siRNA by lipofectamine 300 transfection reagent (Invitrogen, Carlsbad, CA, USA) for 48 h. Inhibition of PEDF mRNA expression were measured by real-time PCR after transfection of cells with PEDF-siRNA. The PEDF silencing did not significantly influence cell viability (>90%).

### Transmission electron microscopy (TEM)

TEM analyses were performed as described previously [17]. In brief, cells were treated with or without H24/R6 and then fixed by 2.5% glutaraldehyde for 2 h at 4°C. After washing with PBS, cells were post-fixed in 1% osmium tetroxide for 2 h, dehydrated in graded acetone, infiltrated and embedded in Epoxy resin. Ultrathin sections of 70 nM were cut in a Leica microtome (Leica RM2165, Japan) and examined by TEM (HITACHI HT-7700, Japan) at an accelerating voltage of 80 kV.

### Statistical analyses

Data are presented as mean ± standard deviation for each group. Data were analyzed by analysis of variance and subsequently by Dunnetts’ test. All statistics were calculated using SigmaStat version 3.5 (Systat Software Inc., Chicago, IL, USA), and a *P* value of less than 0.05 was considered statistically significant.

## Results

### Effects of PEDF overexpression and silencing on H/R-induced ROS in HCM

**Fig 1:**
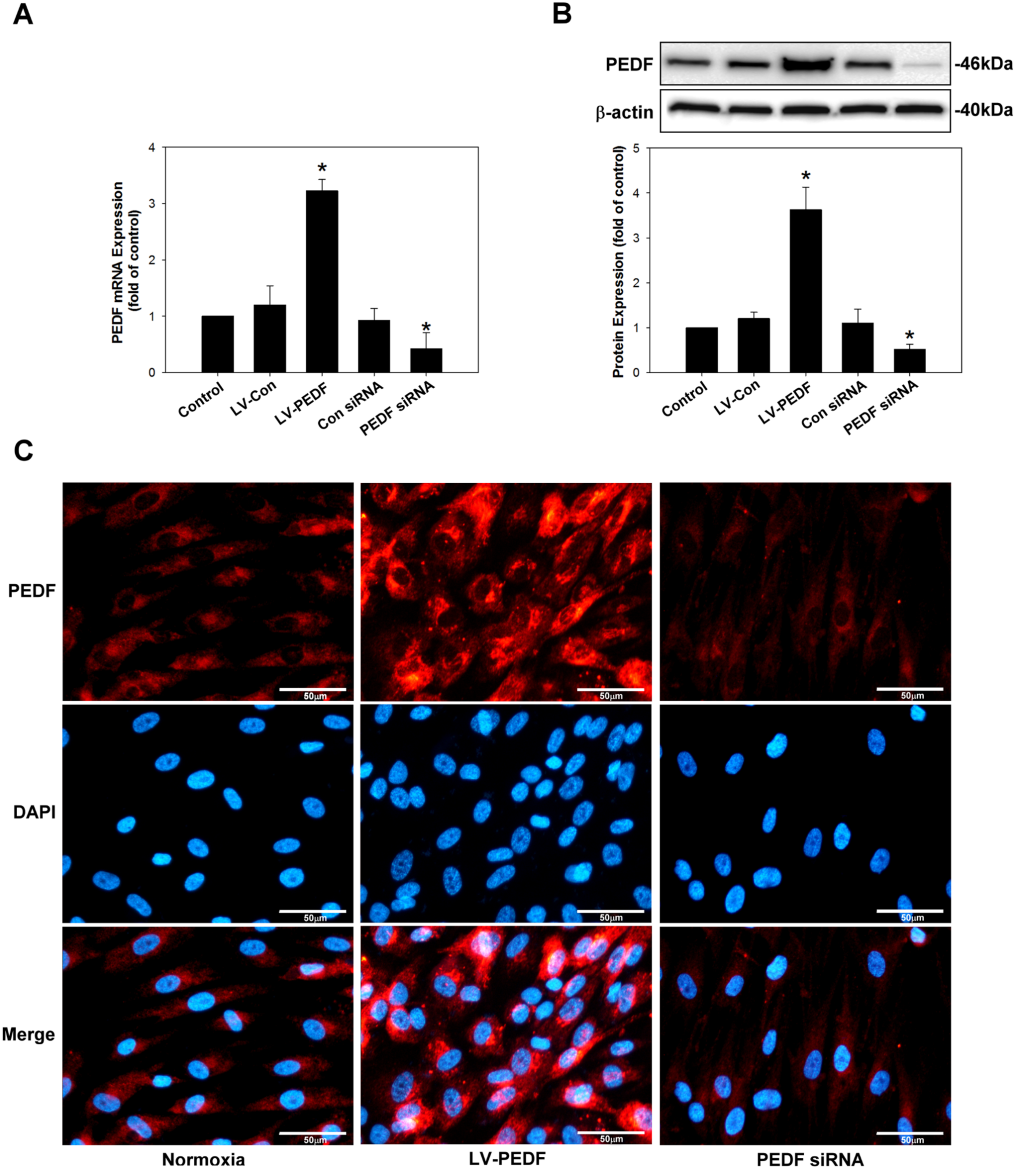
Overexpression or silencing of PEDF expression in HCM. Overexpression or silencing of PEDF mRNA or protein expression were measured by (A) Quantitative PCR, (B) Western blot, and (C) immunocytochemistry analysis, respectively. Data represent the results of three independent experiments. *P < 0.05 versus control condition.

We developed cell lines that stably maintain the hPEDF (LV-PEDF) and pLEX-MCS (empty vector; LV-con) vectors by lentiviral transduction. Meanwhile, PEDF mRNA silencing was achieved by transfecting HCM with the PEDF small interfering RNA (siRNA) plasmid using a lipofectamine reagent. Using these PEDF expression and silencing vectors, we obtained approximately a 3.5–4.0-fold increase, and greater than a 50% decrease in PEDF expression, respectively, as confirmed by real-time PCR (Fig 1A), Western blot (Fig 1B), and immunocytochemistry (Fig 1C) analyses.

**Fig 2:**
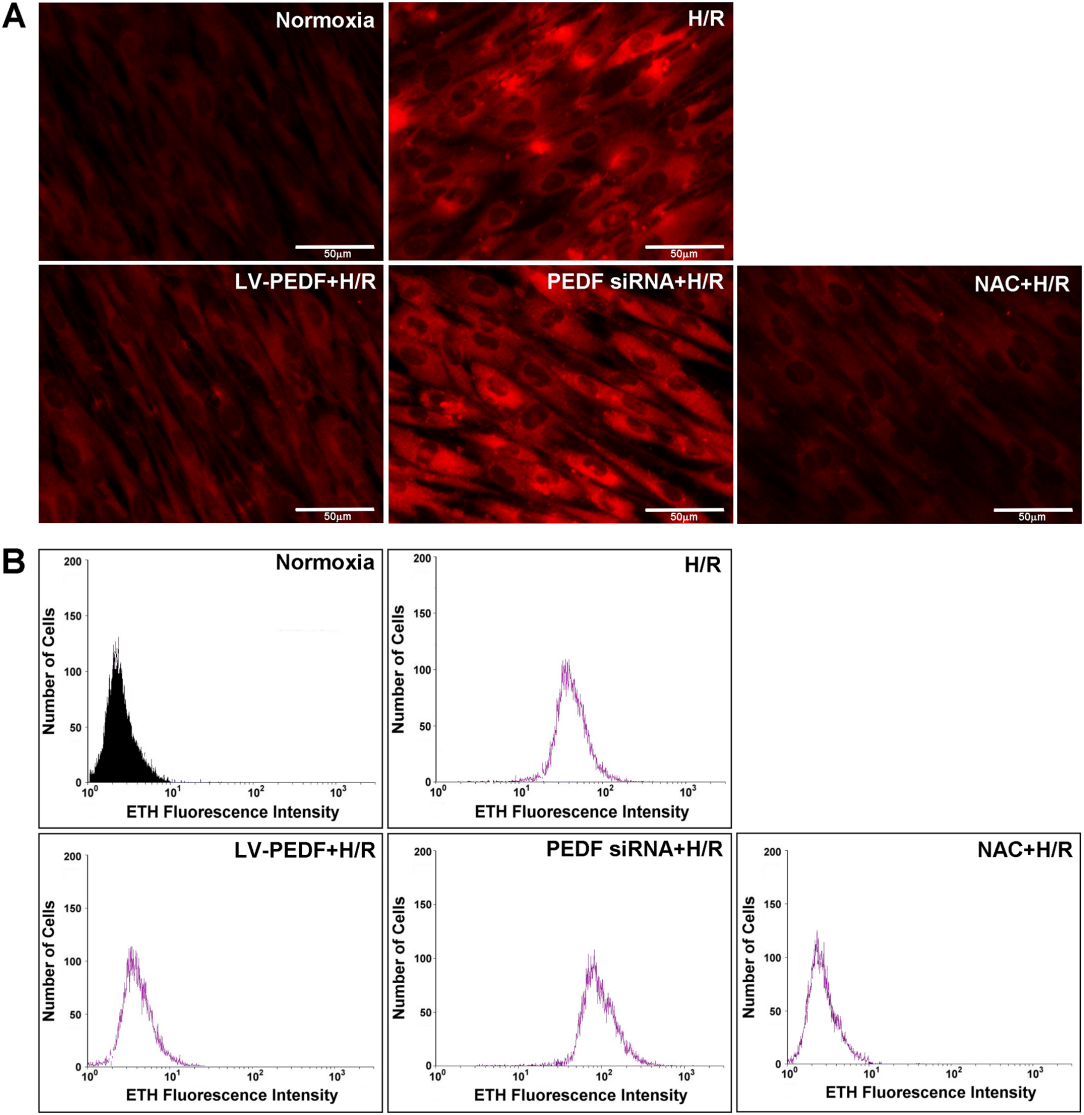
PEDF decreases H/R-induced oxidative stress production in HCM. Levels of intracellular ROS production were detected by (A) fluorescence microscopy and (B) flow cytometry analyses using the ROS-sensitive probe DHE. Data represent the results of three independent experiments.

As described previously [18], H/R injury can trigger ROS production. To test whether PEDF regulates ROS homeostasis under pathophysiological conditions, we measured the effects of HCM, LV-PEDF, PEDF siRNA, and NAC (ROS scavenger) pretreatment on ROS generation in cells subjected to H/R using fluorescent ROS-sensitive probes. As expected, fluorescence microscopy (Fig 2A) and flow cytometry analysis (Fig 2B) of fluorescent probes-detectable ROS revealed an increase in fluorescence intensity in cultured adult cells subjected to H/R. As shown in Fig 2, overexpression of PEDF resulted in a significant decrease in the H/R-induced production of ROS, while cells expressing the PEDF siRNA exhibited enhanced H/R-dependent ROS production compared with that of the H/R alone (control) group. These findings suggest that PEDF exerts a cardioprotective effect by mediating the levels of intracellular ROS generated in response to H/R.

### PEDF reduces H/R-caused mitochondrial dysfunction

**Fig 3:**
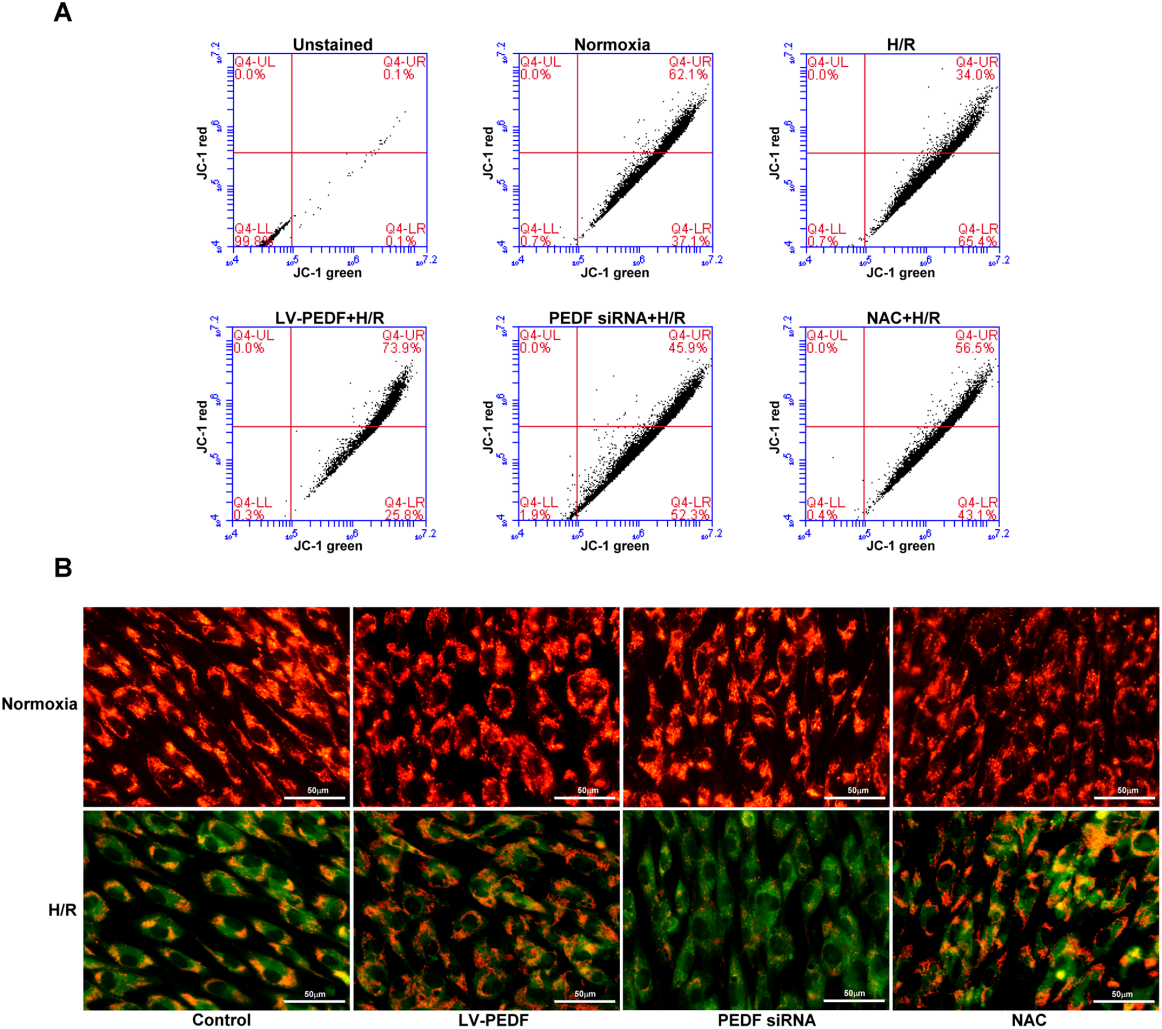
PEDF attenuates H/R-induced mitochondrial *ΔΨm* in HCM. Cells were cultured, exposed to H/R or control conditions, and stained with JC-1 dye. Cells were then subjected to quantitative analysis via (A) flow cytometry and (B) fluorescence microscopy. Loss of *ΔΨm* was determined by measuring changes in JC-1-derived fluorescence from red (at high potential as J-aggregates) to green (at low potential as a monomer) in H/R-treated cells. Data represent the results of three independent experiments.

Mitochondrial dysfunction, which is involved in apoptotic cell death [19], was evaluated *in vitro* by measuring changes in the Δ*Ψ*_m_ of HCM exposed to H/R conditions. For these analyses, live cells were stained with the cationic dye JC-1, and Δ*Ψ*_m_ was evaluated by flow cytometry (Fig 3A) and fluorescence microscopy (Fig 3B). JC-1 exhibits potential-dependent accumulation in mitochondria, indicated by a fluorescence emission shift from red (∼590 nm) to green (∼525 nm). As such, a red JC-1 signal (green color right shift) was representative of the Δ*Ψ*_m_ in the HCM. The peak JC-1 fluorescence intensities measured for the red/green fluorescence at normoxic conditions, and for the H24/R6, LV-PEDF + H24/R6, PEDF siRNA + H24/R6, and NAC + H24/R6 groups were 62.1 ± 5.5% / 37.1 ± 4.8%, 34.0 ± 6.1% / 65.4 ± 4.8%, 73.9 ± 4.0% / 25.8 ± 6.5%, 45.9 ± 3.7% / 52.3 ± 7.5%, and 56.5 ± 3.5% / 43.1 ± 4.8%, respectively. These results indicate that H/R conditions caused comparable levels of Δ*Ψ*_m_ downregulation in both the H/R alone and PEDF siRNA groups, and that overexpression of PEDF successfully attenuated the H/R-induced Δ*Ψ*_m_ depletion.

### PEDF reduces H/R-induced cell apoptosis

**Fig 4:**
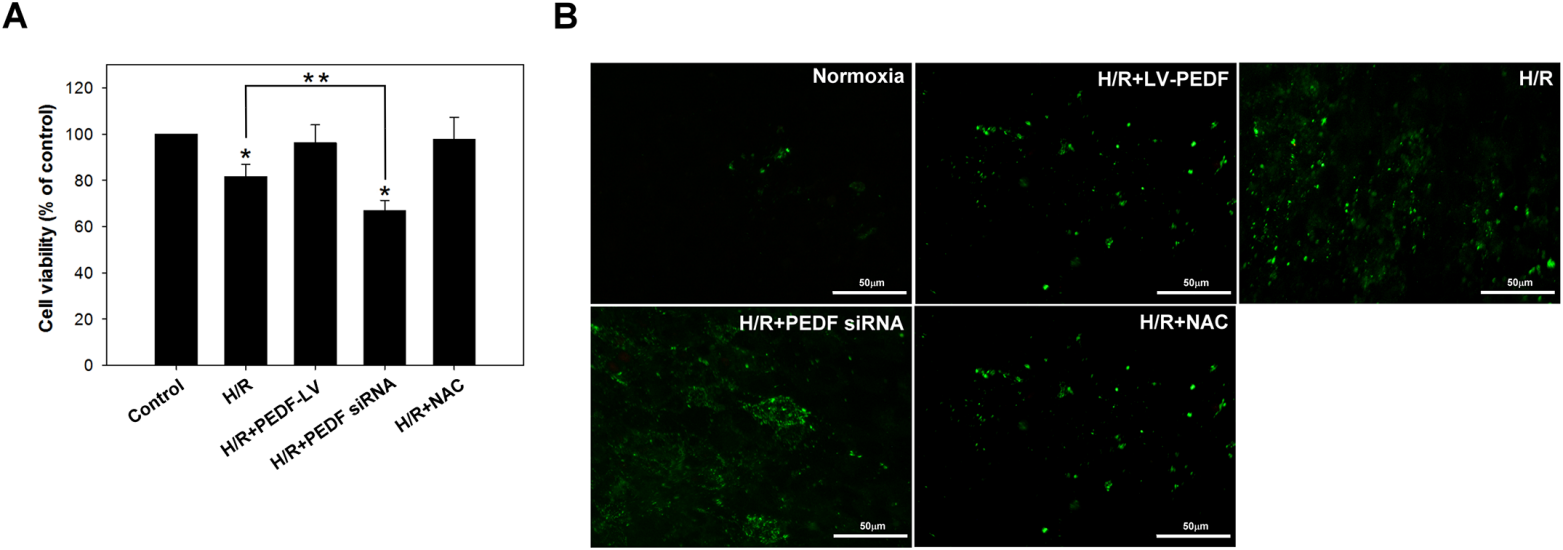
PEDF diminishes H/R-induced cell apoptosis in HCM. HCM and HCM exposed to H/R conditions were stained with annexinV-FITC and subjected to (A) MTT and (B) fluorescence microscopy analyses. Data represent the results of three independent experiments. *P < 0.05 versus control condition. **P < 0.05 versus two treatment groups.

The cytotoxic effects of H/R on HCM were initially assessed by the MTT assay. While the H3/R6 treatment (short-duration hypoxia) did not significantly affect cell viability in any group, the H24/R6 treatment (long-duration hypoxia) significantly reduced cell viability (up to 80.1 ± 2.3%) compared with that of the normoxia group. In addition, overexpression of PEDF attenuated the H24/R6-mediated reduction in cell viability, while silencing of PEDF expression promoted H3/R6-induced cell death (Fig 4A). Subsequently, annexin V staining (Fig 4B) analysis demonstrated that H24/R6 treatment resulted in the induction of apoptosis in HCM, and that this increase in apoptosis was attenuated by overexpression of PEDF. In contrast, silencing of PEDF expression exacerbated the levels of apoptosis under H/R conditions.

**Fig 5:**
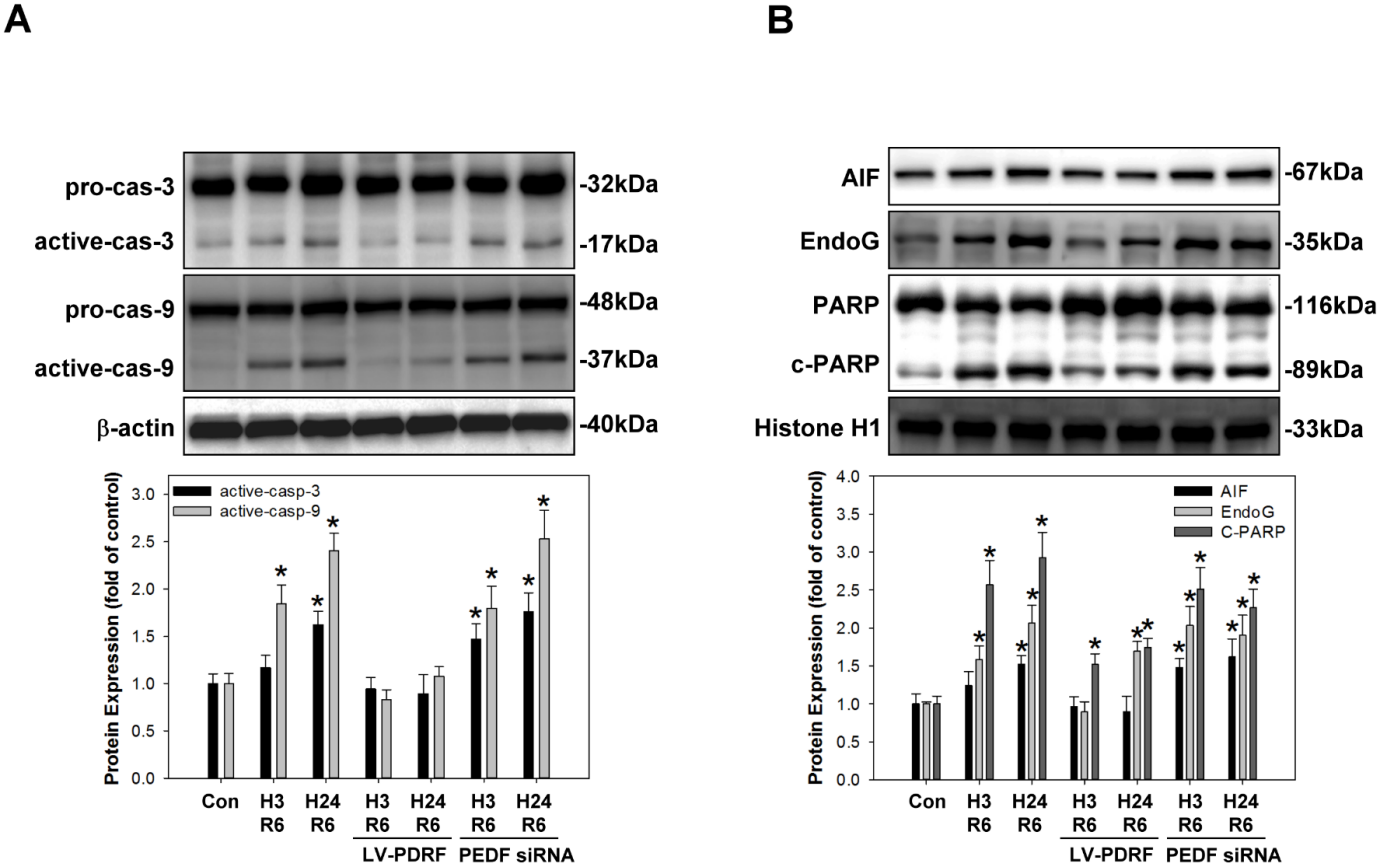
PEDF attenuates H/R-induced expression of pro-apoptotic proteins and activation of caspase-dependent and -independent apoptosis pathways in HCM. Cell were pretreated with rapamycin (100 nM/24 h), 3-MA (2 mM/24 h), or Z-VAD-FMK (25 μM/1 h) and then divided into control and H/R treatment groups. The cytosolic and nuclear proteins were harvested from both treatment groups, and western blot analysis was utilized to evaluate the levels of (A) the caspase-dependent pathway proteins caspase-3 (cas-3) and caspase-9 (cas-9), (B) the caspase-independent pathway proteins AIF and EndoG. Data represent the results of three independent experiments. *P < 0.05 versus control condition.

Mitochondria play important roles in apoptosis by activating the caspase cascade (Fig 5A) and releasing proapoptotic factors, such as AIF, and endonuclease G (EndoG) (Fig 5B), which activate caspase-dependent and independent apoptosis pathways, thereby promoting cleavage of the DNA repair protein PARP (Fig 5B).

### PEDF promotes autophagy

**Fig 6:**
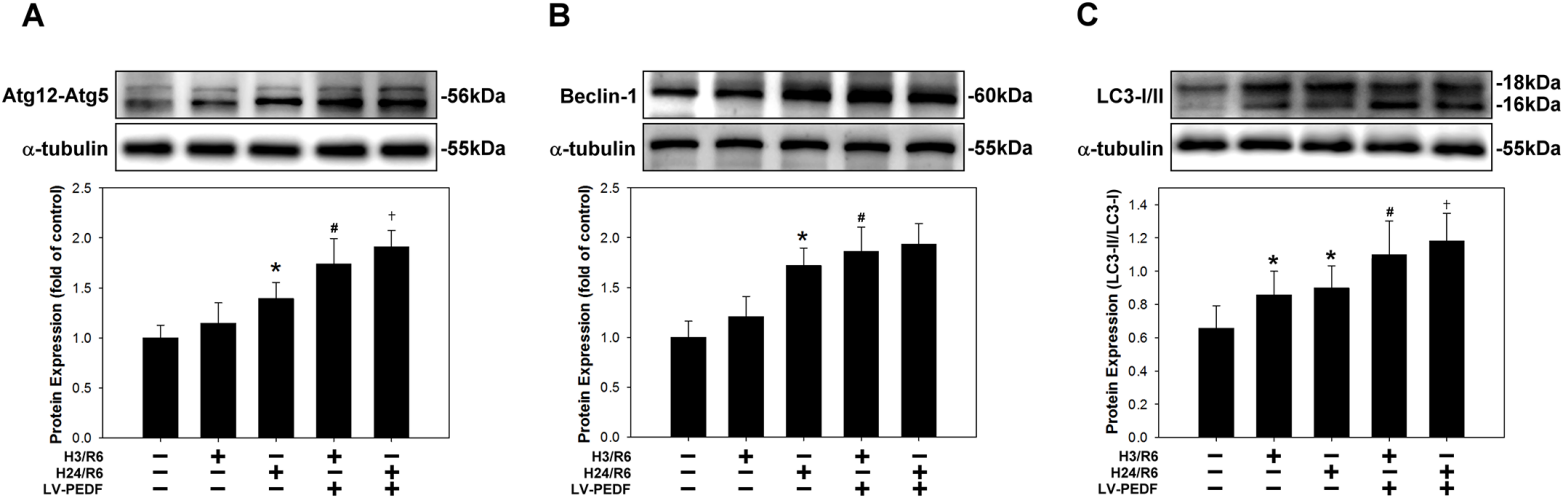
Effects of PEDF over expression on the levels of autophagic indicators. HCM and HCM subjected to H/R treatment were harvested Western blot analysis of the expression levels of the autophagy related proteins (A) Atg12-Atg5, (B) Beclin-1, and (C) and LC3. Data are representative of the results of three independent experiments. *P < 0.05 versus normoxic control group; ^#^*P* < 0.05 versus H3/R6 group; ^†^*P* < 0.05 versus H24/R6 group.

Autophagy promotes cell survival via the catabolism of intracellular resources to maintain bioenergetics under nutrient limiting conditions, and by elimination of damaged organelles and toxic protein aggregates [20]. We speculated that PEDF-mediated macroautophagosome formation/function may contribute to the survival of HCM under H/R conditions. To address this hypothesis, we analyzed the expression levels of the autophagy marker proteins Atg12-Atd5 (Fig 6A), Beclin-1 (Fig 6B), and LC3-I/II (Fig 6C) in HCM under normoxic and H/R (H3/R6 and H24/R6) conditions by Western blot. H/R conditions resulted in significant increases in Atg12-Atd5 and Beclin-1 expression, as well as an increase in the number of LC3 puncta (LC3-I to LC3-II conversion), compared with the normoxic control group. In addition, overexpression of PEDF further significantly increased H/R-caused autophagy flux, compared with their corresponding control groups, H3/R6 and H24/R6, respectively.

**Fig 7:**
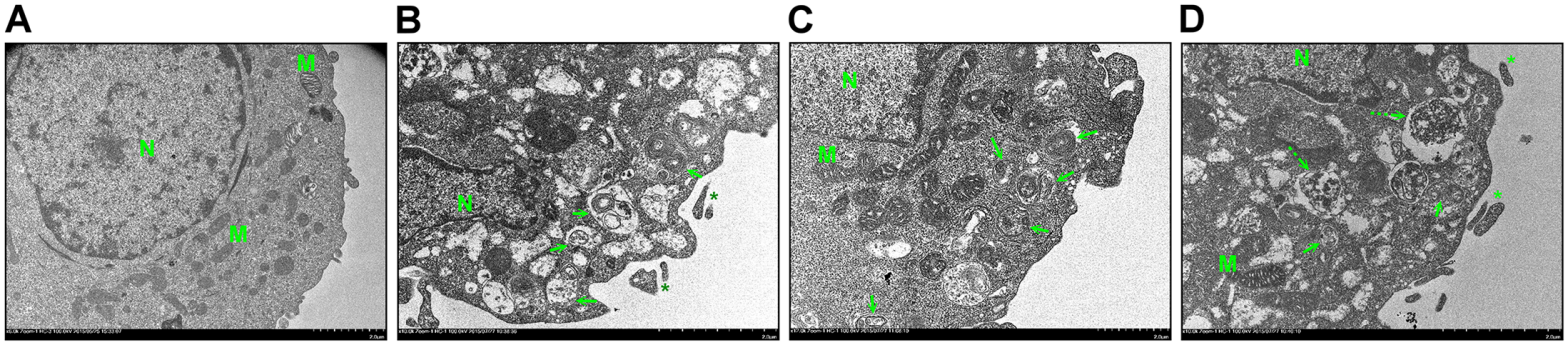
Analysis of ultrastructural changes in HCM after H/R by transmission electron microscopy (TEM). Representative images of TEM analysis of HCM in the (A) normoxia, (B) H/R, (C) H/R+LV-PEDF, and (D) H/R+ PEDF siRNA groups. The abundant vacuoles of various sizes present in cytoplasm of cells in panels B–D were typical autophagosomes (arrow) containing degenerating mitochondria (M). Cell shrinkage and nuclear (N) chromatin condensation, loss of cellular organelles, formation of vacuoles (V), autolysosomes/amphisomes (hatched arrow), and apoptotic bodies (asterisk) were observed in B and D. Data are from three independent experiments.

Next, we attempted to evaluate the effects of PEDF expression on ultrastructural changes in HCM under H/R conditions by TEM (Fig 7). Notably, we detected numerous healthy mitochondria and large autophagic vacuoles with typical double-layered membranes containing organelle remnants in the cells overexpressing PEDF cells, but not in the untreated cells (H/R alone) or in the cells treated with the PEDF-specific siRNA.

## Discussion

In this study, we showed that overexpression of PEDF attenuates H/R-induced cardiomyocyte injury via ROS elimination, inhibition of the mitochondrial-mediated apoptosis pathway, and induction of autophagy. Indeed, consistent with previous reports, our flow cytometry and western blot analyses revealed that H/R conditions induced cellular ROS production, *ΔΨm* depletion, and reduced PEDF levels in cardiomyocytes [5, 8]. Conversely, *in vitro* analyses revealed that PEDF overexpression yielded decreased ROS generation and *ΔΨm* depletion, while promoting autophagy and inhibiting the activation of the mitochondrial-mediated apoptosis pathway under H/R conditions.

The mitochondrial outer membrane permeabilization induces the release of cytochrome *c* from mitochondria into the cytoplasm, and then activates the caspase-9 proenzyme upon H/R condition. Once initiated caspase-9 descend to cleave procaspase-3, an apoptosis-related cysteine peptidase, thereby leading to nuclear chromatin condensation and apoptotic DNA fragmentation [21]. Notably, overexpression of PEDF under H/R conditions reduced caspase-dependent pathway activation and DNA fragmentation. In addition to caspase activation, the release of cytochrome *c* from mitochondria comprises an important step in the regulation of the caspase-dependent apoptotic pathway. Meanwhile, the caspase-independent cell death pathway involves the release of AIF and EndoG from mitochondria, which then translocate into the nucleus to induce chromatin condensation and large-scale DNA fragmentation [22]. In this study, silencing of PEDF expression promoted nuclear translocation of AIF and EndoG, as well as cleavage of PARP and apoptosis, to a greater extent than H/R alone. Previous studies have shown that the protective effects of PEDF under H/R conditions may be due to ROS inhibition, increase in ROS clearance, and/or inhibition of ROS-mediated caspase-dependent and -independent pathways, leading to enhanced cell survival.

H/R increases ROS production leading to apoptosis and autophagy [23]. Autophagy is a rescued mechanism responsible for the continuous headroom of misfolded proteins or unnecessary organelles in lysosomes [24], and for maintaining differentiation, remodeling, and cellular homeostasis [25]. Autophagic and apoptotic cell death comprise the two important forms of H/R-induced cardiomyocyte cell death [26]. In this study, pretreatment of cardiomyocytes with the autophagy inducer rapamycin attenuated H/R-induced caspaspe-3 activation and DNA fragmentation. We also detected significantly increased expression of the autophagtic proteins Beclin 1, Atg-12-Atg5, and LC3II in cardiomyocytes overexpressing PEDF compared to the levels observed in the H/R alone group under H/R conditions. Furthermore, via TEM analyses, we observed significant ultrastructural morphological changes in HCM under H/R conditions, including the production of autophagic vacuoles, autophagosomes containing degenerating mitochondria or other organelles, cell shrinkage, nuclear chromatin condensation, the formation of vacuoles, and apoptotic bodies [27, 28]. In contrast to our findings, other studies indicate that under pathological conditions, autophagy can act as a harmful form of procedural cell death [29]. Indeed, multiple appearance of autophagy, such as an increased number of autophagic vacuoles in human failure cardiomyocyte [30]. Here, cardiomyocytes overexpressing PEDF exhibited homogeneous nuclear chromatin with a small number of agglutinations, and rich organelles within the cytoplasm. Furthermore, these cells exhibited healthy mitochondria that had maintained their structural integrity. In contrast, silencing of PEDF expression resulted in inhibition of autophagic flux and marked increases in the number of autophagic vacuoles and apoptotic bodies. These results indicate that PEDF can enhance H/R-induced autophagy, and therefore suggest that increasing the levels of autophagy may comprise a novel pathway for PEDF-mediated therapy for improving cardiomyocyte injury.

Our study has limitations. It is unclear what is the molecular mechanism behind PEDF-modulated anti-oxidant and anti-apoptotic factors, and how to promote autophagy in H/R injury. Moreover, in our study, PEDF was only 50% silenced is of concern in the interpretation since the remaining 50% could influence the direction of change of apoptosis and autophagy. Finally, in the TEM data, it would be beneficial for a quantitative methodology could be employed such as a blinded scoring system evaluating multiple cells in each treatment group would be better. It is important to explore the effective mechanisms for PEDF in the further directions for the field of research H/R-related heart injury.

In the present study, we demonstrated that H/R treatment resulted in increased production of ROS, and the ROS stress-mediated induction of the mitochondria-related apoptosis pathways, including autophagy and cell apoptosis. Upregulation of autophagy and downregulation of the caspase-dependent and -independent apoptosis pathways occurred as an effective protective cellular response against H/R-induced cell death. Thus, our observations support those of recent reports suggesting that PEDF alleviates H/R-induced ROS production, thereby inhibiting apoptosis and promoting cell autophagy. Furthermore, they indicate that enhancing PEDF expression may comprise a potential therapeutic approach for H/R-induced myocardial injury.
